# Optimizing a machine learning based glioma grading system using multi-parametric MRI histogram and texture features

**DOI:** 10.18632/oncotarget.18001

**Published:** 2017-05-18

**Authors:** Xin Zhang, Lin-Feng Yan, Yu-Chuan Hu, Gang Li, Yang Yang, Yu Han, Ying-Zhi Sun, Zhi-Cheng Liu, Qiang Tian, Zi-Yang Han, Le-De Liu, Bin-Quan Hu, Zi-Yu Qiu, Wen Wang, Guang-Bin Cui

**Affiliations:** ^1^ Department of Radiology, Tangdu Hospital, The Fourth Military Medical University, Xi’an 710038, Shaanxi, P.R. China; ^2^ Department of Neurosurgery, Tangdu Hospital, The Fourth Military Medical University, Xi’an 710038, Shaanxi, P.R. China; ^3^ Student Brigade, The Fourth Military Medical University, Xi’an 710032, Shaanxi, P.R. China

**Keywords:** glioma grading, MRI, machine learning, attribute selection, support vector machine (SVM)

## Abstract

Current machine learning techniques provide the opportunity to develop noninvasive and automated glioma grading tools, by utilizing quantitative parameters derived from multi-modal magnetic resonance imaging (MRI) data. However, the efficacies of different machine learning methods in glioma grading have not been investigated.A comprehensive comparison of varied machine learning methods in differentiating low-grade gliomas (LGGs) and high-grade gliomas (HGGs) as well as WHO grade II, III and IV gliomas based on multi-parametric MRI images was proposed in the current study. The parametric histogram and image texture attributes of 120 glioma patients were extracted from the perfusion, diffusion and permeability parametric maps of preoperative MRI. Then, 25 commonly used machine learning classifiers combined with 8 independent attribute selection methods were applied and evaluated using leave-one-out cross validation (LOOCV) strategy. Besides, the influences of parameter selection on the classifying performances were investigated. We found that support vector machine (SVM) exhibited superior performance to other classifiers. By combining all tumor attributes with synthetic minority over-sampling technique (SMOTE), the highest classifying accuracy of 0.945 or 0.961 for LGG and HGG or grade II, III and IV gliomas was achieved. Application of Recursive Feature Elimination (RFE) attribute selection strategy further improved the classifying accuracies. Besides, the performances of LibSVM, SMO, IBk classifiers were influenced by some key parameters such as *kernel type*, *c*, *gama*, *K*, etc. SVM is a promising tool in developing automated preoperative glioma grading system, especially when being combined with RFE strategy. Model parameters should be considered in glioma grading model optimization.

## INTRODUCTION

Gliomas are the most common brain tumors all over the world and can be classified into different grades, i.e. low-grade gliomas (LGGs) including grade I and grade II as well as high-grade gliomas (HGGs) including grade III and grade IV, according to World Health Organization (WHO) criteria. Preoperative glioma grading is crucial as the therapeutic strategies are quite disparate for different grades, which may further influence the patient's prognosis [1–3]. Pathological diagnosis after biopsy or surgery is predominately used as the gold standard. However, the inevitable sampling error and invasive procedure may bring more risks than benefits to glioma patients. Moreover, this histological examination is usually time-consuming [4, 5], challenging timely glioma grading.

Recently, researchers devoted to exploring a non-invasive neuroimaging tool for glioma grading by using diverse quantitative parameters derived from advanced magnetic resonance imaging (MRI) techniques, such as dynamic contrast enhanced MRI (DCE-MRI) [2, 3, 6], arterial spin labeling (ASL) [7, 8] and diffusion weighted imaging (DWI) [9–11]. Despite various correlations between parameter features (or attributes) and glioma grades reported in the literature, considerable difficulties emerge when selecting the imaging biomarkers with the best accuracy and reproducibility. Moreover, even for one single modal MRI, it is still not decided which features contribute most to diagnosis, those from commonly used histogram parameters or image texture attributes [2, 3, 9, 10, 12–14]? Thus, feature selection is an unsolved critical issue and should be carefully performed when making the preoperative glioma grading.

Facing tons of information offered with multimodal MRI, selecting the most effective features and coming to the satisfying diagnostic accuracy with mankind is a big challenge. With the development of artificial intelligence technology, machine learning techniques are gradually applied in glioma imaging studies [6, 15, 16]. Compared with previous receiver operating characteristics (ROC) diagnostic analysis, machine learning demonstrates several advantages [7, 9]. First, a subset of vital features that contribute most or are most relevant to glioma grading can be picked up with suitable feature selection methods [4, 17]. Furthermore, the machine can automatically learn the discrimination patterns from the existing data and establish the corresponding model to predict the individual glioma grade [16, 18]. Additionally, the classifying model can be further optimized to improve its diagnostic accuracy by selecting an appropriated classifier, optimizing model parameters or specific validation procedure [4, 19, 20]. Thus, it is expected to develop a high-efficient machine learning based glioma grading system utilizing informative multi-parametric MRI features.

Even so, varied machine learning classifiers, feature selection strategies and model parameters unavoidably introduced difficulties to determine the glioma grading model, making the optimization work critically important. Thus, in the current study we first constructed a comprehensive machine learning based glioma grading system using the combined parametric histogram features and image texture attributes of multi-parametric tumor images, and then tried to achieve the overall optimal grading model by investigating the influence of different feature selection strategies and classification methods on the performances of glioma grading. We aimed to provide an effective preoperative glioma grading tool with the best use of the multi-parametric MRI images.

## RESULTS

### Demographical and clinical results

The statistical results of the demographical and clinical characteristics of LGG and HGG patients involved in our experiment were summarized in Table 1. It was suggested that there was no significant group difference between LGG patients and HGG patients on gender and tumor location except for age (P<0.001). The pathological types for each grade gliomas were summarized in Supplementary Table 1

**Table 1:** Baseline demographics and clinical characteristics of patients

Variable	LGG (grade I/II)	HGG (grade III/IV)	P value
**Patients**	N=28	N=92	-
**Gender^a^**			
Male	46.4% (13/28)	57.6% (53/92)	0.298
Female	53.6% (15/28)	42.3% (39/92)	
**Age^b^**			
Mean±SD	35.9±16.2	49.3±15.0	<0.001*
**Location^c^**			
Supratentorial	10.7%(3/28)	1.1%(1/92)	0.060
Subtentorial	89.3%(25/28)	98.9%(91/92)	
**Histologic feature**			
Diffuse astrocytoma	39.3%(11/28)	-	-
Oligodendroglioma	7.1%(2/28)	-	-
Oligoastrocytoma	39.3%(11/28)	-	-
Anaplastic astrocytoma	-	6.5%(6/92)	-
Anaplastic oligodendroglioma	-	5.4%(5/92)	-
Anaplastic oligoastrocytoma	-	19.6%(18/92)	-
Glioblastoma	-	67.4%(62/92)	NA
Miscellaneous	14.3%(4/28)	1.1%(1/92)	NA

Note: Difference between LGG and HGG patients was evaluated with the Pearson Chi-Square^a^, unpaired Student t test^b^ and continuity correction^c^. * The difference between the LGG and HGG groups was significant.

### Multi-parametric MRI images

**Figure 1:**
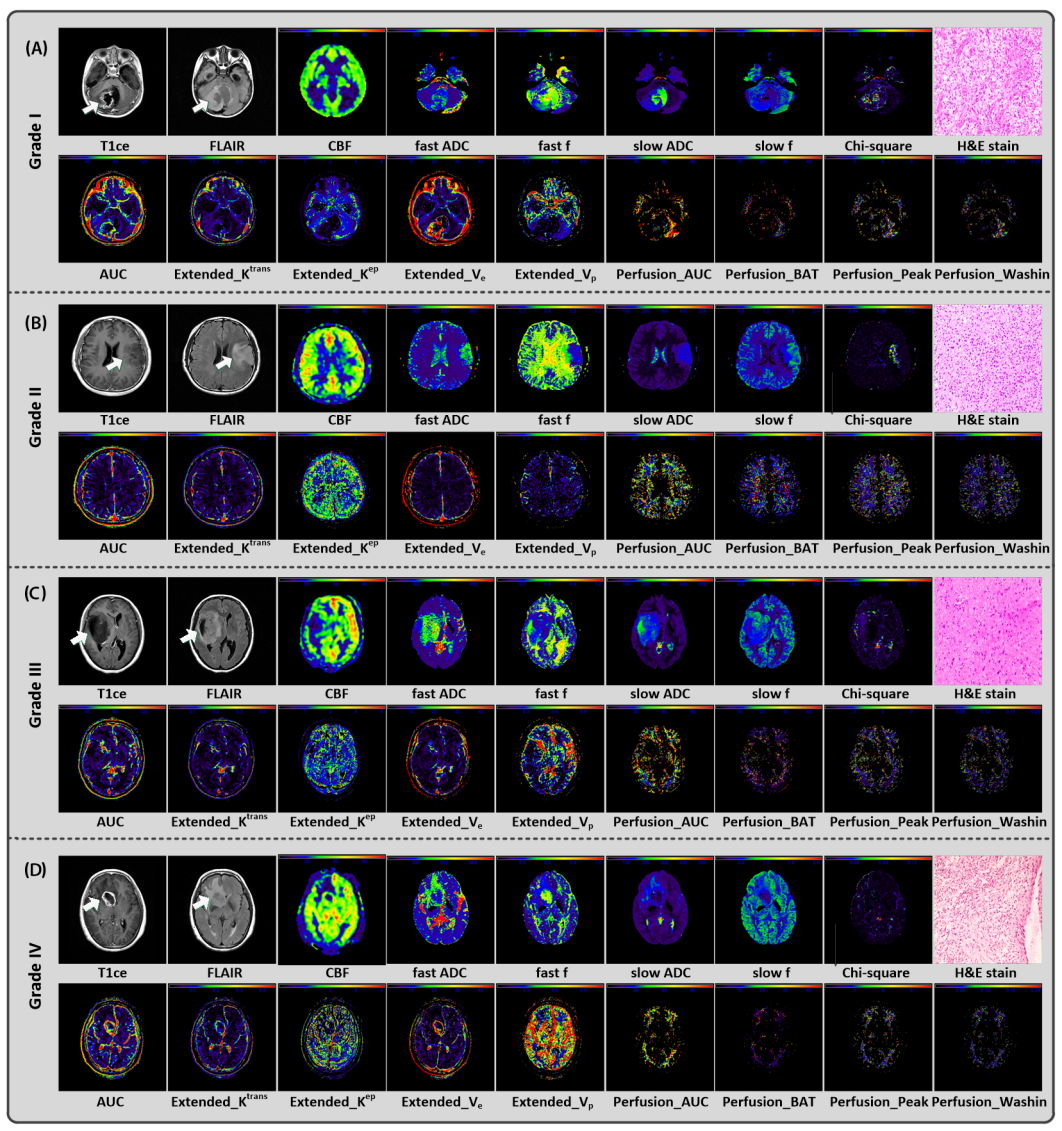
Conventional/multi-parametric MRI maps and H&E stain results of 4 individuals diagnosed as grade I **(A)**, II **(B)**, III **(C)**, and IV **(D)** gliomas, respectively. For each individual, 1 parametric map derived from 3D ASL (i.e. CBF), 5 parametric maps derived from multi b-value DWI (i.e. fast ADC, fast f, slow ADC, slow f and Chi-square maps), part of parametric maps derived from DCE (9 out of 24, i.e. AUC_AIF_, Extended_K^rans^, Extended_K^ep^, Extended_V_e_, Extended_V_p_, Perfusion_AUC_FP_ Perfusion_BAT, Perfusion_Peak, and Perfusion_Washin) and H&E stain (i.e. haematoxylin and eosin) result were shown.

The example conventional, multi-parametric images and pathological haematoxylin and eosin (H&E) stain results of four individual patients diagnosed of WHO grade I, grade II, grade III and grade IV were provided in Figure 1. For each individual, conventional MRI images (T1ce/FLAIR), ASL parametric map (CBF), DWI parametric maps (fast ADC, fast *f*, slow ADC, slow *f* and Chi-square) and part of DCE parametric maps (9 out of 24 parameters, i.e. AUC_AIF_, “Extended_K^trans^, Extended_K^ep^, Extended_V_e_, Extended_V_p_, Perfusion_AUC_EP_” Perfusion_BAT, Perfusion_Peak, and Perfusion_Washin) were figured for the selected slice with glioma. The H&E stain results demonstrated that the HGG gliomas (grade III and grade IV) had relatively high cell density (see Supplementary Figure 1).

After multi-parametric MRI histogram and texture attribute extraction and collection, the imbalanced tumor attribute samples were preliminarily oversampled with SMOTE [17] and a newly normalized attribute combination composed of 100 LGG and 100 HGG samples was generated (as shown in Table 2). Similarly, to discriminate the grade II, III and IV gliomas, each class was oversampled to new datasets with 68 samples in each grade.

**Table 2:** The classification accuracy/AUC of 25 WEKA classifiers using combined multi-parametric histogram and texture attributes in LGG and HGG as well as grade II, III and IV gliomas classification

25 WEKA classifiers (accuracy/AUC)		LGG vs. HGG		Grade II, III and IV	
		Original (28 vs. 92)	Smote (100 vs. 100)	Original (25 vs. 29 vs. 63)	Smote (68 vs. 68 vs. 68)
**Bayes**	BayesNet	0.717/0.743	0.750/0.826	0.667/0.836	0.770/0.880
	NaiveBayes	0.742/0.717	0.845/0.874	0.641/0.778	0.750/0.885
**Lazy**	**IBk^##^**	0.750/0.638	0.905/0.905	0.718/0.795	**0.961^##^**/0.971
	LWL	0.733/0.769	0.800/0.833	0.735/0.858	0.642/0.892
**Functions**	LibSVM (linear)^** #^	0.792/0.690	0.945^*^/0.945	**0.786^#^**/0.838	0.956/0.957
	Logistic	0.708/0.698	0.885/0.934	0.556/0.716	0.828/0.895
	SimpleLogistic	0.792/0.841	0.930/0.957	0.735/0.888	0.922/0.976
	SGD	0.792/0.715	0.930/0.930	0.701/0.816	0.917/0.977
	SMO^**#^	0.758/0.668	0.945^*^/0.945	**0.786^#^**/0.874	0.956/0.975
	VotedPerceptron	0.758/0.697	0.800/0.861	0.590/0.758	0.657/0.873
**Meta**	AdaBoostM1*	0.808*/0.793	0.875/0.956	0.675/0.925	0.809/0.894
	Bagging	0.783/0.818	0.855/0.933	0.726/0.912	0.858/0.966
	ClassificationViaRegression	0.708/0.800	0.830/0.900	0.658/0.879	0.939/0.843
	LogitBoost*	0.808*/0.846	0.885/0.945	0.675/0.891	0.877/0.974
**Rules**	Decision Table	0.642/0.597	0.795/0.896	0.761/0.912	0.745/0.871
	Jrip	0.767/0.612	0.850/0.808	0.726/0.838	0.814/0.879
	OneR	0.792/0.616	0.645/0.645	0.718/0.809	0.672/0.754
	PART	0.633/0.567	0.830/0.804	0.692/0.821	0.775/0.818
**Trees**	DecisionStump	0.767/0.629	0.815/0.630	0.726/0.771	0.304/0.608
	HoeffdingTree	0.742/0.218	0.850/0.875	0.650/0.777	0.750/0.885
	J48	0.675/0.397	0.855/0.801	0.684/0.817	0.833/0.872
	LMT	0.800/0.849	0.930/0.958	0.744/0.896	0.922/0.976
	RandomForest	0.792/0.845	0.915/0.976	0.752/0.892	0.922/0.984
	RandomTree	0.658/0.540	0.815/0.813	0.607/0.698	0.755/0.818
	REPTree	0.742/0.460	0.820/0.850	0.650/0.837	0.779/0.901

*, ** represent the classifier with the highest classifying accuracy on original and SMOTE LGG and HGG glioma data, respectively. #, ## represent the classifier with the highest classifying accuracy on original and SMOTE grade II-III-IV glioma data, respectively.

### Preliminary comparison among 25 WEKA classifiers

Linear kernel was initially used for LibSVM classifier, regarding that linear SVM is qualified for big attribute number condition and default parameters were used for all the classifiers. The classifying performance without attribute selection was preliminarily summarized in Table 2.

It was revealed that the highest classifying accuracy was 0.808 using LogitBoost (AUC=0.846) and AdaBoostM1 (AUC=0.793) classifiers for raw LGG and HGG data. The other classifiers showed much lower accuracy, implying the lower potential of clinical application. However, these results were not reliable due to severe imbalance of original data (with low AUC values). Based on the new dataset generated with SMOTE, almost every classifier exhibited significant improvement of classifying performance, except for OneR classifier. The highest classifying accuracy reached 0.945 by using LibSVM or SMO classifier, both of which were SVM classifiers.

Similar results were revealed in classifying grade II, III and IV gliomas. The highest accuracy was only 0.786 (SMO classifier with AUC = 0.874 and LibSVM classifier with AUC = 0.838) for original samples, yet it increased to 0.956 along with increased AUC (0.957 for LibSVM classifier and 0.975 for SMO classifier) using SMOTE samples. The highest performance was acquired by using IBk classifier with accuracy = 0.961 and AUC =0.971. Thus, the following investigations and comparisons were performed on SMOTE datasets.

### Classification comparison with attribute selection

**Figure 2:**
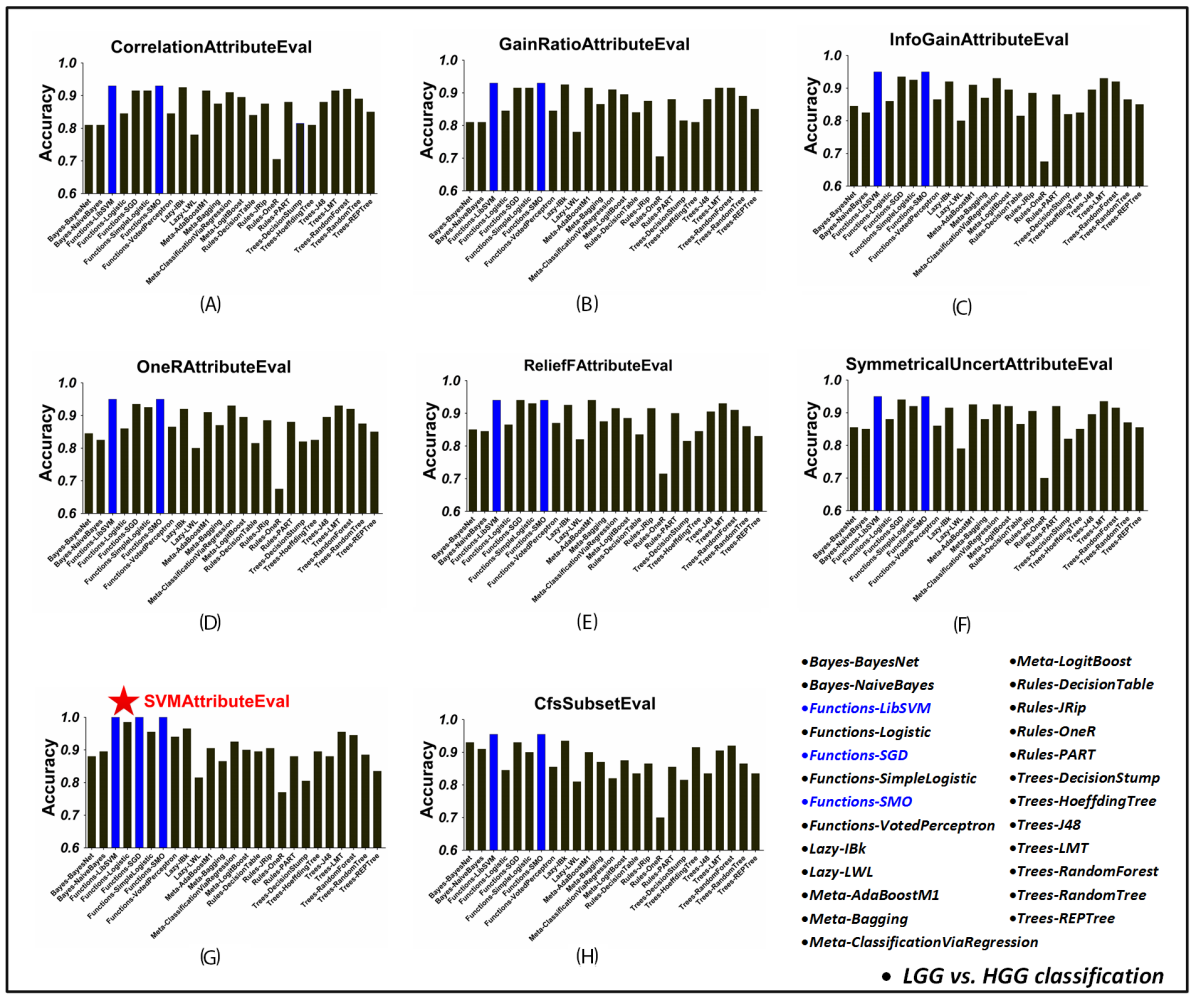
The classification accuracy of 25 WEKA classifiers in LGG and HGG classification, using each attribute selection strategy **(A)–(G)** Using ‘CorrelationAttributeEval’, ‘GainRatioAttributeEval’, ‘InfoGainAttributeEval’, ‘OneRAttributeEval’, ‘ReliefFAttributeEval’, ‘SymmetricalUncertAttributeEval’ and ‘SVMAttributeEval’ with ‘Ranker’ search method, respectively. **(H)** Using ‘CfsSubsetEval’ with ‘BestFirst’ search method. Under each attribute selection strategy, the highest accuracy among all the 25 WEK. In each figure, blue bars mean the highest classification accuracy across classifiers using the corresponding attribute selection method. The overall best result was achieved when using ‘SVMAttributeEval’ attribute slection method with LibSVM/SGD/SMO classifiers as shown in **(G)**.

**Figure 3:**
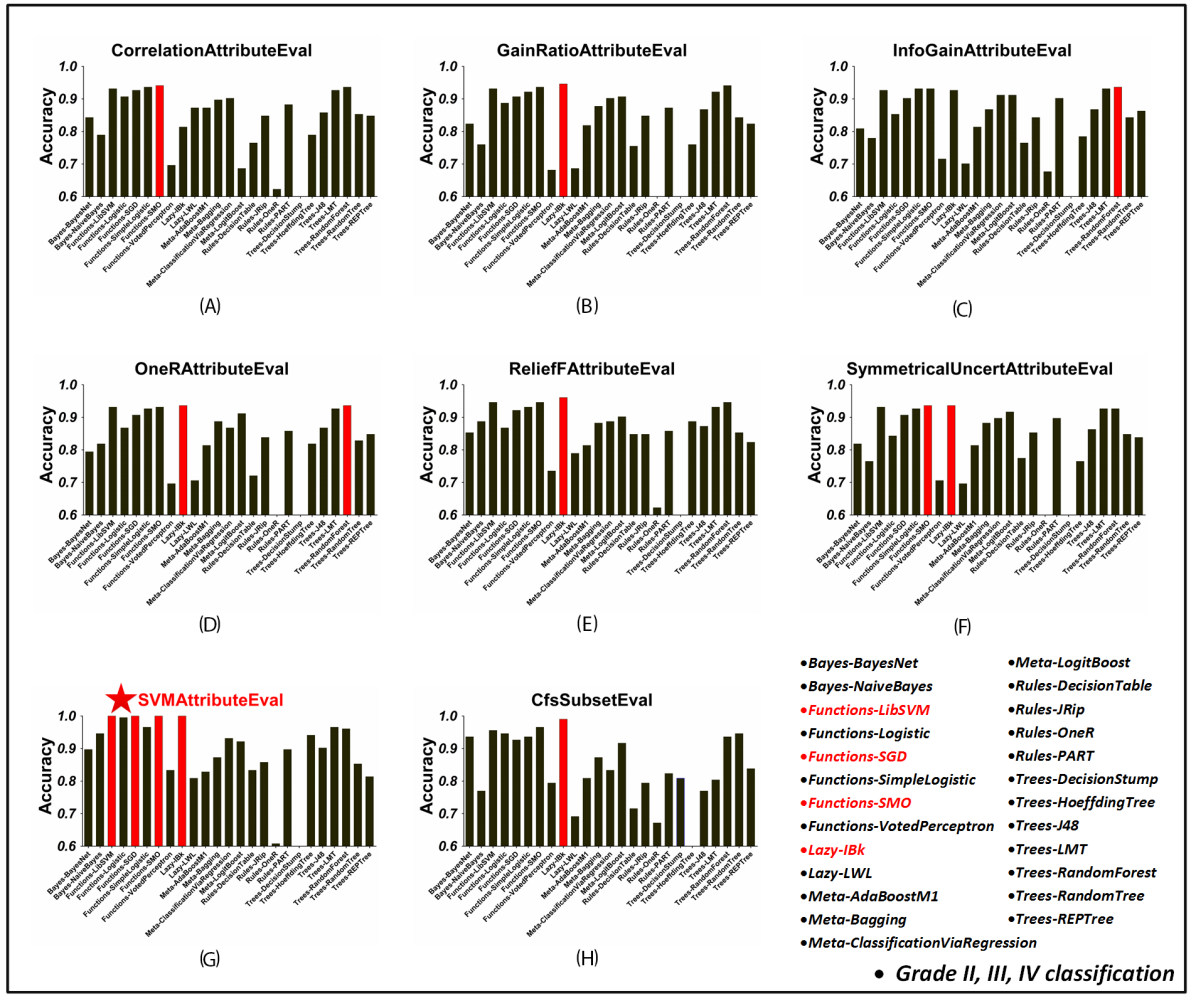
The classification accuracy of 25 WEKA classifiers in grade II, III and IV gliomas classification, using each attribute selection strategy **(A)~(G)** Using ‘CorrelationAttributeEval’, ‘GainRatioAttributeEval’, ‘InfoGainAttributeEval’, ‘OneRAttributeEval’, ‘ReliefFAttributeEval’, ‘SymmetricalUncertAttributeEval’ and ‘SVMAttributeEval’ with ‘Ranker’ search method, respectively. **(H)** Using ‘CfsSubsetEval’ with ‘BestFirst’ search method. In each figure, red bars mean the highest classification accuracy across classifiers using the corresponding attribute selection method. The overall best result was achieved when using ‘SVMAttributeEval’ attribute slection method with LibSVM/SGD/SMO/IBk classifiers as shown in (G).

The tumor attributes were independently re-ranked according to the rank outcome using seven ranking metrics. The top 50~600 attributes with a stepwise of 50-attribute in each ranking sequence were selected to test classifying accuracies for each classifier. The classification performances using different numbers of top-ranked attributes were investigated for each classifier and the highest accuracy was recorded as its optimal value under the corresponding ranking strategy. On the other hand, by applying the ‘CfsSubsetEval’ method, the best first attributes were sorted out. Based on this attribute subset, the classification results of each classifier were obtained. After that, all the classifiers were compared across attribute selection methods. The optimal classifying accuracy of these classifiers under each attribute selection strategy in discriminating LGG and HGG gliomas as well as grade II, III and IV gliomas were visualized in Figures 2 and 3, respectively.

It turned out that in LGG and HGG glioma classification, both LibSVM and SMO classifiers got top accuracy for each attribute selection situation (Figure 2). The best result was achieved when combined with ‘SVMAttributeEval’ ranking method, i.e. SVM Recursive Feature Elimination (SVM-RFE) method. Besides, the SGD, IBk, AdaBoostM1, LMT and RandomForest classifiers also exhibited superior performance to others with high accuracy over 0.9. As shown in Figure 3, grade II, III and IV glioma classification got similar results. In spite of different top classifiers under each attribute selection strategy (including IBk, RandomForest, SMO, LibSVM, etc.), the overall best result was achieved when using ‘SVMAttributeEval’ evaluating method combined with SMO/LibSVM/SGD/IBk classifiers. All of the above results suggested the high performance of jointly using SVM classifier and SVM-RFE attribute selection method in glioma grading.

The top ranked attributes in ‘SVMAttributeEval’ sequence were further surveyed here. We found that the highest accuracy have already reached up to 1 for SMOTE LGG and HGG samples when using top 50 attributes combined with SMO and LibSVM classifiers. Twenty-three out of them came from texture analysis and other 27 attributes were from histogram analysis of multi-parameter data. It was observed that CBF (derived from ASL), D* and *D* (derived from multi b-values DWI), K^ep^, K^trans^, V_e_ and perfusion parameters including AUC_FP_, peak-value, and wash-out time (derived from DCE-MRI) held the majority of top important attributes (37 out of 50). Extended TOFTs model was superior to other three models. As for grading II, III and IV gliomas, the top 50 attributes, i.e. 25 histogram attributes and 25 texture attributes, were a bit different from those for classifying LGG from HGG gliomas. They mainly covered the following parameters: D* from DWI, K^ep^, V_e_, V_p_, perfusion AUC_FP_ and peak-value from DCE-MRI. Similarly, Extended TOFTs model outperformed other models. The details of the top 50 SVM-RFE attributes selected in LGG and HGG classification as well as grade II, III and IV classification were listed in Supplementary Table 2.

### Model parameter selection

**Figure 4:**
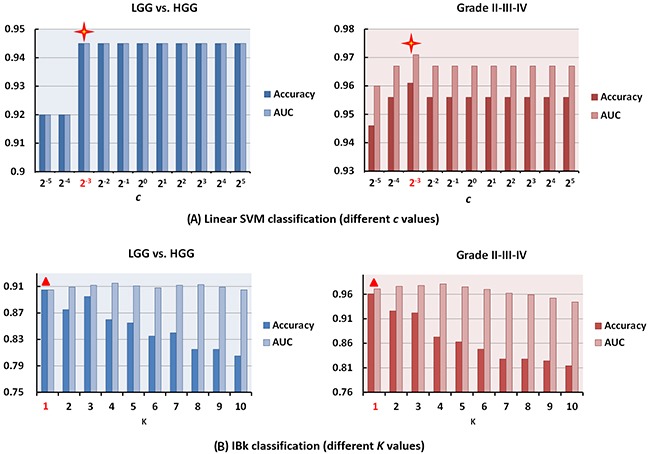
The influence of key model parameters for linear SVM and IBk classifiers **(A)** The classification performance of LibSVM (linear) classifier using different *c*. When using *c*=2^-3^, the best classification performance was achieved for both LGG and HGG (Accuracy/AUC = 0.945/0.945) as well as grade II, III, and IV (Accuracy/AUC = 0.961/0.971) gliomas classification. **(B)** The classification accuracy and AUC values of IBk classifiers using different *K* in *KNN* for LGG and HGG as well as grade II, III, IV gliomas classification, respectively.

Three high-efficient classifying models, i.e. SMO, LibSVM and IBk classifiers were discussed in this section based on the original attribute collection. First, linear kernel and RBF kernel were independently analyzed for LibSVM classifier. For linear LibSVM, different *c* values were applied and the classification performances were compared (Figure 4A). It was revealed that *c*=2^-3^, but not the default value (*c*=1) is the best parameter for our purpose. For RBF LibSVM, different combinations of varied *c* and *gamma* were investigated in Table 3. When using *gamma*=2^-6^ and *c*=2^1^ for LGG and HGG data or *gamma*=2^-7^ and *c*=2^3^ for grade II, III and IV glioma data, the highest accuracy and AUC values were achieved (default: *gamma*=0 and *c*=1).

**Table 3:** The classification performance of LibSVM (RBF) classifier using different *c* and *gamma*

Accuracy/AUC		*c*=2^-5^	*c*=2^-4^	*c*=2^-3^	*c*=2^-2^	*c*=2^-1^	*c*=2^0^	*c*=2^1^	*c*=2^2^	*c*=2^3^	*c*=2^4^	*c*=2^5^
**LGG vs. HGG**	*gamma*=2^-8^	0/0	0.685/0.685	0.730/0.730	0.745/0.745	0.815/0.815	0.880/0.880	0.930/0.930	0.940/0.940	0.945/0.945	0.950/0.950	0.950/0.950
	*gamma*=2^-7^	0.280/0.280	0.725/0.725	0.745/0.745	0.765/0.765	0.850/0.850	0.930/0.930	0.950/0.950	0.950/0.950	0.950/0.950	0.950/0.950	0.950/0.950
	***gamma*=2^-6^**	0.270/0.270	0.720/0.720	0.750/0.750	0.810/0.810	0.900/0.900	0.940/0.940	**0.955**/0.955	0.955/0.955	0.955/0.955	0.955/0.955	0.955/0.955
	*gamma*=2^-5^	0/0	0.595/0.595	0.755/0.755	0.805/0.805	0.885/0.885	0.945/0.945	0.950/0.950	0.950/0.950	0.950/0.950	0.950/0.950	0.950/0.950
	*gamma*=2^-4^	0/0	0/0	0.525/0.525	0.800/0.800	0.830/0.830	0.895/0.895	0.900/0.900	0.900/0.900	0.900/0.900	0.900/0.900	0.900/0.900
	*gamma*=2^-3^	0/0	0/0	0/0	0/0	0.690/0.690	0.815/0.815	0.835/0.835	0.835/0.835	0.835/0.835	0.835/0.835	0.835/0.835
	*gamma*=2^-2^	0/0	0/0	0/0	0/0	0/0	0.690/0.690	0.710/0.710	0.710/0.710	0.710/0.710	0.710/0.710	0.710/0.710
	*gamma*=2^-1^	0/0	0/0	0/0	0/0	0/0	0.120/0.120	0.155/0.155	0.155/0.155	0.155/0.155	0.155/0.155	0.155/0.155
**Grade II, III, and IV**	*gamma*=2^-8^	0/ 0.250	0.235/0.426	0.265/0.449	0.534/0.651	0.770/0.827	0.843/0.882	0.922/0.941	0.946/0.960	0.971/0.978	0.975/0.982	0.971/0.978
	***gamma*=2^-7^**	0.005/0.254	0.265/0.449	0.309/0.482	0.765/0.824	0.838/0.879	0.912/0.934	0.946/0.960	0.971/0.978	**0.981**/0.985	0.981/0.985	0.981/0.985
	*gamma*=2^-6^	0/ 0.250	0.265/0.449	0.588/0.691	0.784/0.838	0.853/0.890	0.941/0.956	0.961/0.971	0.966/0.974	0.966/0.974	0.966/0.974	0.966/0.974
	*gamma*=2^-5^	0/ 0.250	0.196/0.397	0.598/0.699	0.789/0.842	0.892/0.919	0.951/0.963	0.961/0.971	0.961/0.971	0.961/0.971	0.961/0.971	0.961/0.971
	*gamma*=2^-4^	0/ 0.250	0/ 0.250	0.078/0.309	0.750/0.813	0.838/0.879	0.838/0.879	0.907/0.930	0.907/0.930	0.907/0.930	0.907/0.930	0.907/0.930
	*gamma*=2^-3^	0/ 0.250	0/ 0.250	0/ 0.250	0/ 0.250	0.466/0.599	0.824/0.868	0.828/0.871	0.828/0.871	0.828/0.871	0.828/0.871	0.828/0.871
	*gamma*=2^-2^	0/ 0.250	0/ 0.250	0/ 0.250	0/ 0.250	0/ 0.250	0.603/0.702	0.652/0.739	0.652/0.739	0.652/0.739	0.652/0.739	0.652/0.739
	*gamma*=2^-1^	0/ 0.250	0/ 0.250	0/ 0.250	0/ 0.250	0/ 0.250	0.054/0.290	0.074/0.305	0.074/0.305	0.074/0.305	0.074/0.305	0.074/0.305

Then, the other two key parameters, *c* and *kernel*, were considered in SMO model and the classification results along with their variations were summarized in Table 4. Compared to default models using *PolyKernel* and *c*=1, the classifying accuracy had a slight increase of 0.015 for both LGG and HGG classification as well as grade II, III and IV glioma discrimination by using *RBFKernel* and *c*=2^2^/2^3^. The AUC values showed similar results.

**Table 4:** The performance of WEKA SMO classifier using different *c* and *kernel*

Accuracy/AUC		*c*=2^-5^	*c*=2^-4^	*c*=2^-3^	*c*=2^-2^	*c*=2^-1^	*c*=2^0^	*c*=2^1^	c=2^2^	c=2^3^	*c*=2^4^	*c*=2^5^
**LGG vs. HGG**	NormalizedPoly-Kernel	0.030/0.030	0.705/0.705	0.735/0.735	0.760/0.760	0.810/0.810	0.865/0.865	0.920/0.920	0.940/0.940	0.950/0.950	0.950/0.950	0.950/0.950
	PolyKernel	0.920/0.920	0.940/0.940	0.945/0.945	0.945/0.945	0.945/0.945	0.945/0.945	0.945/0.945	0.945/0.945	0.945/0.945	0.945/0.945	0.945/0.945
	**RBFKernel**	0.390/0.390	0.725/0.725	0.745/0.745	0.795/0.795	0.865/0.865	0.930/0.930	0.955/0.955	**0.960/0.960**	0.960/0.960	0.960/0.960	0.960/0.960
	PUK	0.295/0.295	0.310/0.310	0.300/0.300	0.265/0.265	0.235/0.235	0.765/0.765	0.745/0.745	0.745/0.745	0.745/0.745	0.745/0.745	0.745/0.745
**Grade II, III, and IV**	NormalizedPoly-Kernel	0.103/0.141	0.456/0.632	0.436/0.626	0.613/0.732	0.775/0.852	0.843/0.900	0.922/0.957	0.941/0.965	0.966/0.981	0.966/0.981	0.966/0.981
	PolyKernel	0.946/0.966	0.956/0.977	0.961/0.979	0.956/0.975	0.956/0.975	0.956/0.975	0.956/0.975	0.956/0.975	0.956/0.975	0.956/0.975	0.956/0.975
	**RBFKernel**	0.186/0.310	0.456/0.629	0.598/0.732	0.770/0.846	0.848/0.909	0.917/0.955	0.956/0.977	0.966/0.983	**0.971/0.986**	0.971/0.986	0.971/0.986
	PUK	0.162/0.350	0.172/0.326	0.152/0.321	0.152/0.309	0.176/0.319	0.745/0.822	0.735/0.819	0.735/0.819	0.735/0.819	0.735/0.819	0.735/0.819

For IBk classifier, the important parameter *K* in KNN was investigated. The best *K* was 1 for our LGG and HGG (accuracy/AUC = 0.905/0.905) data as well as grade II, III and IV (accuracy/AUC = 0.961/0.971) glioma data (Figure 4B).

All the above results demonstrated the importance of optimizing model parameters for machine learning based glioma grading studies.

## DISCUSSION

In summary, we proposed a comprehensive automated glioma grading scheme integrating advanced multi-parametric MRI data with machine learning methods. Various commonly used classifiers and attribute selection approaches were conducted in order to optimize the most effective machine learning tool for preoperative glioma grading. SVM is proved to be superior to the other classifiers, and achieved the best performance when combined with RFE attribute selection strategy. In addition, the selection of some key model parameters, such as *kernel type*, *gamma*, *c* in SVM models, *K* in IBk model, etc., may influence the classifier's performance. The current study suggested the importance of classifier type, attribute selection methods and model parameters in auto-grading of gliomas using machine learning techniques.

The analysis flow of generating multi-parametric MRI maps, extracting and selecting effective tumor attributes as well as optimizing machine learning models offered the opportunity to establish the comprehensive non-invasive preoperative glioma grading system. To our knowledge, it is the first report to inspect the performance of commonly used machine learning methods for glioma grading. Inevitably, there are some limitations for the present study. The classification accuracy of the proposed machine learning glioma grading system seemed very high (over 90%) in the current study, probably override experienced neuro-radiologist. This could be real owing to the great contributions of multi-parametric attributes and effective machine learning techniques, or could be associated with the following factors to some extent. First, our patient data were biased across glioma grades, i.e. more HGG (especially grade IV) samples than LGG ones. The oversampling procedure with SMOTE was applied and the performance of grading models were largely improved after that. However, the SMOTE procedure only generated new datasets from original data and the minority samples were oversampled even more than three times of the original data, which might not fully represent the features of the minority class (i.e. LGG). Thus, this operation may result in a model with relatively high classification accuracy on current data but bad performance on new dataset. Second, the over-fitting risk of machine learning could not be avoided by cross-validation procedure. More independent testing dataset should be collected to further test the performances of models. Moreover, the applied LOOCV method in this study repeatedly used the original samples during each training and testing procedure. It was not recommended for larger dataset than the current one. More generalized validation approaches and strategies should be performed on large datasets in the future. In addition, the classifiers inspected in this study did not embrace all the classification techniques; specially, the deep learning was not included, which is a powerful tool for representing big and complex data [21].

Despite that multi-parametric MRI images were investigated in previous glioma grading studies, most of them have been focused on analyzing the relationship between the parameter values and glioma grades and evaluating their discriminating ability using conventional ROC method. However, it is difficult to determine which parameter and parameter feature is the best for glioma grading and it is impractical for accurately individualized diagnosis. According to previous studies, various MRI parameters can reflect the glioma grading information in distinct aspects, e.g. DCE-derived permeability parameters such as K^trans^ [2, 3, 14], V_e_ [3], V_p_ [2], and etc., DWI-derived diffusion parameters including ADC [9, 11], *D* [9], D* [9], and ASL-derived perfusion CBF [7] parameter were all considered to be helpful in distinguishing the differences between different grade gliomas, however, some of them were found to be not significantly correlated with glioma grades in some studies [2, 22, 23]. Thus, it is much possible that not one single parameter but the comprehensive parametric combination affords the most effective discriminative ability. Thus, instead of using one specific parameter, we collected multi-modal MRI parametric images and automatically selected the most effective and informative parameter combinations for glioma grading through proper attribute selection techniques.

Recently, machine learning approaches have been applied in diagnostic studies of various cancers such as prostate cancer [17], breast cancer [24], lung cancer [25], colorectal cancer [26], gliomas [15], etc. The good performance and the potential clinical application value of machine learning were concerned, typically in the radiomics studies utilizing the diverse imaging data [25, 26]. Our results also indicated that the machine learning approach using multi-parametric MRI attributes can help to improve the predictive performance of glioma grading. Thus, it is expected to explore a set of automated cancer diagnosis systems in the future. Whereas, there are still some blocks to reach this goal. Though various machine learning algorithms were proposed, each of them had inherit advantages and disadvantages. Thus, it's difficult to select the optimal approach for the complex cancer data. On the other hand, the current machine learning based method depended mostly on the technique itself. The variation of model parameters or samples may lead to an obvious variation of model performance. A big amount of samples will be needed for improving the stability and generalization ability of the trained models before clinical application. What's more, the influence of the complex and diverse data collected from different imaging devices with inconsistent parameters in different institutions should also be carefully considered. Meanwhile, the attribute extraction and attribute selection procedures could also be very complicated. Then, it will be hard to say which kinds of attributes from what kinds of data were the optimal for diagnosis expect for a large number of experiments. All in all, it will be a promising but challenging way to the extensive application of machine learning in cancer diagnosis.

This study provided evidence for establishing a high-efficient and accurate automated preoperative glioma grading system. By data mining on the big patient data using optimal classification model with the improved automatic tumor segmentation procedure, a valuable computer-aided preoperative glioma grading system is very promising and feasible for clinic use in the near future. This system will largely assist the clinicians to make appropriate treatment plans and improve the prognosis of glioma patients.

As discussed above, we will try to improve in the following aspects in our future research. First, a large number of balanced sample data will be introduced in model construction to avoid the imbalanced sample problem. Second, two-fold cross validation strategy and further validation on samples collected from independent institutions will be performed to improve the model's generalization ability. Finally, deep learning technique will be integrated into our study, in order to automatically exploit the potentially advanced discriminative tumor features and classify the glioma grades with higher performance. It is expected to play a superexcellent role in glioma grading.

## MATERIALS AND METHODS

**Figure 5:**
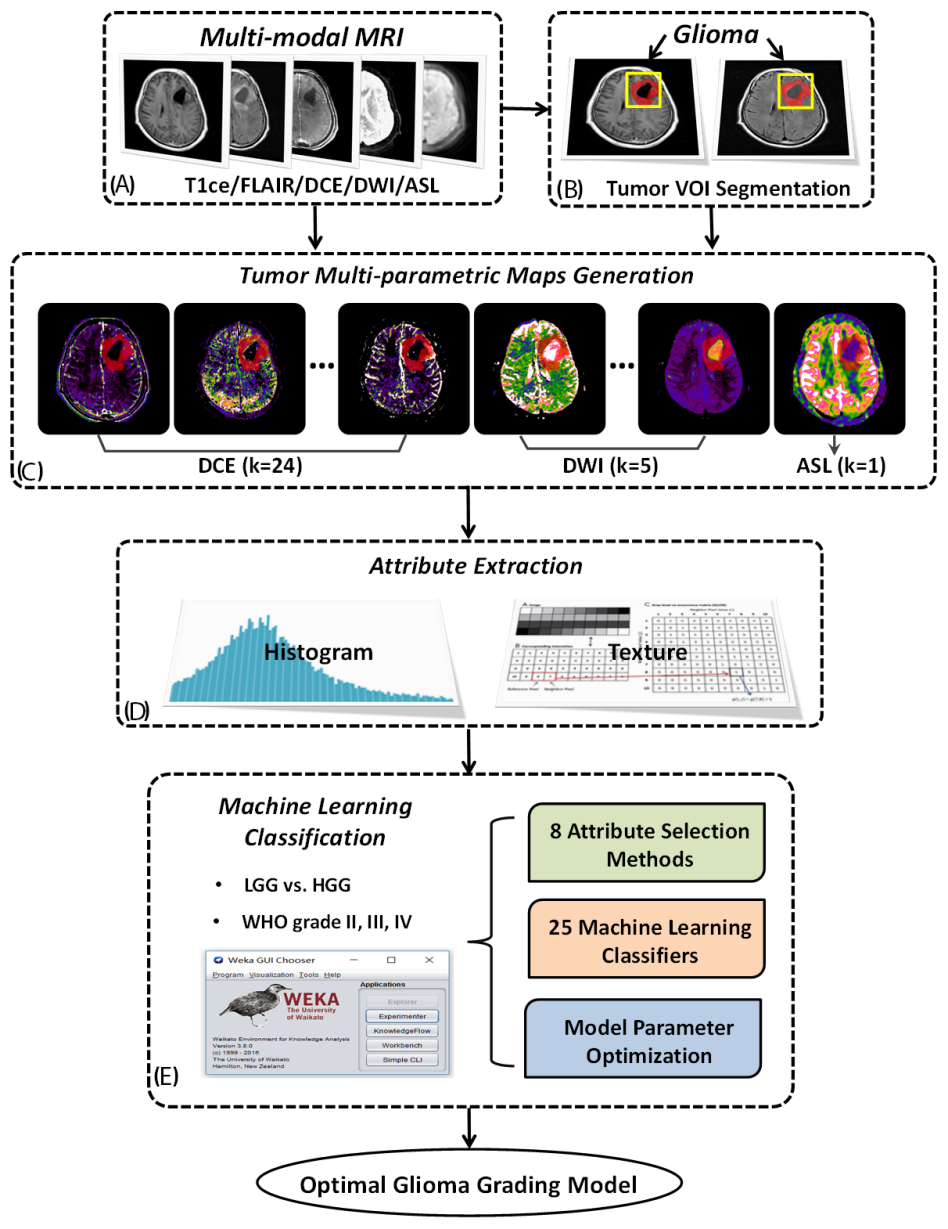
The flowchart of the current study Based on multi-modal MRI data including DCE-MRI, multi-b DWI and 3D-ASL **(A)** and tumor volume of interest (VOI) manually drawn on resampled T1ce or FLAIR image **(B)**, a group of permeability, diffusion and perfusion parametric images were derived and the corresponding parametric maps of the whole tumor region were extracted **(C)**. Utilizing histogram analysis and texture analysis, a big collection of tumor parameter attributes was acquired for the following machine learning process **(D)**. 25 commonly used classifiers and 8 attribute selection methods were implemented and compared using WEKA software with additional discussion on model parameters to construct the optimal glioma grading model **(E)**.

The study data of the current project derived from a diagnostic trial that has been registered to ClinicalTrials.gov (NCT02622620, https://www.clinicaltrials.gov/) with the trial protocol published [27]. The overall analysis scheme was described in Figure 5 on how to integrate the histogram and textual attributes (i.e. features) (Supplementary Table 3) of multi-parametric MRI images into pattern classification methods. Briefly, a group of permeability, diffusion and perfusion related parametric images were first generated from DCE-MRI, DWI and ASL scanning. Then, using parametric histogram and image texture analyses, a number of tumor attributes were extracted from each parametric map within the tumor region. The essence of this study is to conduct a set of machine learning classifications and feature selection methods using Waikato Environment for Knowledge Analysis (WEKA) software [4] in combination with model parameter evaluation, to optimize the most effective classifying model for glioma grading. It is noted that two kinds of classifying tasks were investigated in this study, i.e. LGG and HGG classification as well as WHO grade II, III and IV classification.

### Patient selection and image acquisition

A total of 120 histologically confirmed glioma patients were enrolled, involving 28 LGGs (3 grade I, 25 grade II) and 92 HGGs (29 grade III, 63 grade IV), approved by the Ethics Committee of Tangdu Hospital of the Fourth Military Medical University (TDLL-20151013). Written informed consent was obtained from all individuals. Each participant underwent preoperative conventional and advanced MRI scans on a 3.0T MRI scanner (Discovery 750, GE Healthcare, Milwaukee, WI, USA) with an 8-channel head coil.

Conventional MRI scans included pre-contrast axial T1-weighted spin-echo imaging (T1WI), contrast enhanced T1WI (T1ce) and pre-contrast fluid attenuated inversion recovery (FLAIR) imaging. Imaging parameters for T1WI/T1ce were TR/TE = 1750 ms/24 ms, slice thickness = 5 mm, slice spacing = 1.5 mm, acquisition matrix = 256×256, field of view (FOV) = 240×240 mm^2^, number of excitation (NEX) = 1; for FLAIR: TR/TE = 8000 ms/165 ms, slice thickness = 5 mm, slice spacing = 1.5 mm, acquisition matrix = 256×256, FOV = 240×240 mm^2^, NEX = 1.

Advanced MRI scans included three dimensional ASL (3D-ASL), multi b-value DWI and DCE-MRI in transverse planes. 3D-ASL and multi b-value DWI were conducted prior to the contrast agent injection, and then DCE-MRI sequences were performed and followed with T1ce. The parameters for 3D-ASL were: TR/TE = 4632 ms/10.5 ms, slice thickness = 4 mm, slice spacing = 0 mm, image matrix = 512×512, FOV = 240×240 mm^2^, NEX = 3, Post label Delay = 1525. Multi b-value DWI is a diffusion weighted echo-planar sequence applied with a single shot spin-echo using 13 different b-values (0~3500 s/ mm^2^) [10]. The corresponding imaging parameters were: TR/TE = 3000 ms/Minimum, slice thickness = 5 mm, slice spacing = 1.5 mm, acquisition matrix = 128×128, FOV = 240×240 mm^2^, NEX = 3. The total scan lasted 5 min and 45 s. DCE-MRI was performed with a dynamic gradient-echo T1, with the following parameters: TR/TE = 2.9 ms/1.3 ms, flip angle = 12°, FOV = 240×240 mm^2^, slice thickness = 2.5 mm, slice spacing = 0 mm, acquisition matrix = 128×128. Fifty phases with a temporal resolution of 4 s were conducted resulting in a total acquisition time of 3 min and 20 s. Gadodiamide contrast agent (CA, 0.5 mmol, 0.2 ml/kg, Omniscan, GE Healthcare, Co. Cork, Ireland) was administered at the rate of 2 ml/s at the end of the fifth phase, followed with a bolus injection of 15 ml saline.

### Parametric image generation and tumor segmentation

A set of permeability, diffusion and perfusion parameters could be calculated from advanced 3D-ASL, multi-b values DWI and DCE-MRI data. Given that lots of parameters were reported to provide valuable information in glioma grading [3, 7, 9], as many parameter maps as possible were generated and considered in this study (see Supplementary Table 3).

NordicICE software (Version 4.0; NordicNeuroLab, Bergen, Norway) was used here to derive multi-parametric maps from DCE and DWI images. First, DCE-MRI data were processed to acquire a serial of pharmacokinetic parameter maps [28] by using four computational models, i.e. TOFTs model, Extended TOFTs model, PATLAK model and Incremental model integrated in the DCE module of NordicICE. Quantitative parameters reflecting the exchange procedure of the physiological CA between the blood plasma (BP) and the extracellular extravascular space (EES), i.e. the CA from BP into EES (K^trans^) or from EES back to BP (K^ep^), the fractional volumes of BP (V_p_) and EES (V_e_), and the area under the curve of the arterial input function (AUC_AIF_) were fully or partly inferred from above models based on the population-based arterial input function (AIF) and a fixed T1 with 1000 ms [28]. Furthermore, perfusion parameters including time to peak (TTP), cerebral blood flow (CBF), wash-in time, wash-out time, peak value, bolus arrived time (BAT) and first pass AUC (AUC_FP_) were also estimated. Besides, parameter maps derived from different models were automatically coregistered using rigid transformation by maximization of mutual information. Then, a total of 24 DCE parametric maps were generated from DCE-MRI for each subject and the detailed parameter names can be found in Supplementary Table 3. The multi b-value DWI images were analyzed using the Intra-voxel Incoherent Motion (IVIM) imaging model in NordicICE [9, 10]. Several diffusion related parameters including the slow apparent diffusion coefficient (ADC), (i.e. *D*), fast ADC (i.e. D*), slow fractional ADC (i.e. slow *f*), and fast fractional ADC (i.e. fast *f*) were calculated and chi-square map was obtained as well. As for 3D-ASL, the CBF parametric map was created based on the GE post-processing platform (FuncTool 4.6) [7].

In total, 30 parametric images were finally generated from DEC-MRI, multi-b value DWI MRI and 3D-ASL data. Since most of them (24 out of 30) came from DCE-MRI images and DCE-MRI contained more slices than T1ce or FLAIR, conventional MRI images (T1ce/FLAIR) were resampled to DCE images using NordicICE software to assure that most of original parametric values were kept. The volume of interest (VOI) for each tumor was manually drawn on the resampled T1ce or FLAIR maps, covering the whole tumor region while excluding the obvious necrosis and edema. Then, it was overlapped on DCE-derived parametric maps and the parameter values within the whole tumor volume were extracted. Furthermore, the pre-drawn VOIs were resampled to DWI-parametric and ASL-CBF maps to obtain the resulting parametric values of the tumor.

### Multi-parametric attribute extraction

For each parametric map of the tumor VOI, two types of features, i.e. histogram attributes [29] and texture attributes [17], were extracted based on the MATLAB platform. More than one thousand tumor attributes were collected in this section and the detailed name of the parametric map and the attributes were listed in Supplementary Table 3.

#### Histogram attributes

Using the parameter value of each pixel within the tumor VOI, twenty-three histogram statistical indictors were measured according to their mathematical definitions [29]. They were: mean, median, mode, standard deviation, variance, standard error of mean (SE-mean), skewness, kurtosis, minimum, maximum, Inter-Quartile Range (IQR), the 25^th^/75^th^ percentile (Q1/Q3), the 10^th^/90^th^ percentile, the 5^th^/95^th^ percentile, the mean of the top five percent data (larger than the 95^th^ percentile), the mean of the low five percent data (lower than the 5^th^ percentile), energy and entrophy, the peak height of the parameter histogram and the corresponding parameter value at the peak point (1000 bins).

#### Texture Attributes

One online texture analysis tool named “radiomics” written in MATLAB code was introduced to conduct image texture analysis (https://github.com/mvallieres/radiomics). Thirty-two gray levels were chosen to rescale each parameter map into gray-level image according to its intensity. The first-order texture attributes (i.e., global attributes) were calculated from the gray histogram distributions, including the variance, skewness and kurtosis. Then, three kinds of 3-dimensional second-order texture analysis based on Gray-Level Co-occurrence Matrix (GLCM) [17], Gray-Level Run-Length Matrix (GLRLM) [30], and Gray-Level Size Zone Matrix (GLSZM) [31] models were independently performed to utilize corresponding indictors such as correlation, energy, variance, dissimilarity and etc. The detailed definition of the four texture models were summarized in Supplementary Table 4. A total of 37 texture attributes were acquired from each parametric map.

### Machine learning techniques

Based on the tumor attributes, diverse classifying methods were carried out to train glioma grading models using WEKA (version 3.8.0) [4]. WEKA is an open-source and powerful machine learning tool with operable GUI interfaces, which assembled lots of popular classifying techniques and is easy-to-use. Three modules containing ‘Preprocess’, ‘Classify’ and ‘Select attributes’ modules were involved to execute data preprocessing, classification and attribute selection operations on the collected tumor attribute dataset. 25 commonly used classifying approaches in combination with 8 different attribute selection strategies were conducted in this study.

#### Data preprocessing

Before classification, one important issue was noticed that the glioma data was highly biased across grades in our experiment, i.e. 28 vs. 92 for LGG and HGG classification, and 25 vs. 29 vs. 63 for WHO grade II, III and IV classification. This imbalanced sampling may bias the trained model to favor the class with majority samples, thereby resulting in that most testing samples were designated into the big class to achieve relatively high accuracy but low sensitivity or specificity [17]. The predicting ability of the learned classifier in this condition is really poor and could not be generalized to new datasets. One solution to solve this problem was sample augmentation, i.e. generating new samples of the minority class by over-sampling. Synthetic minority over-sampling technique (SMOTE) [17] was generally recommended (also supported in ‘WEKA-Preprocess’ module). Before that, each attribute of individual patients was normalized to 0~1 according to the minimal and maximal values among all subjects.

#### Attribute selection

Attribute (i.e. feature) selection is of vital importance for classification [4, 17, 32]. A huge number of multi-parametric attributes were retrieved in this study, some of which may play essential roles in glioma grading while the others may be negative or completely useless for glioma grading. Thus attribute selection is critical to sort the most effective attribute subset and improve the classifying ability. Several commonly used attribute selection methods were integrated in the ‘Select attributes’ module in WEKA. Among them, eight were employed in the current study to optimize attribute selection, including seven distinct attribute ranking strategies and one for selecting the best attributes. The ranking programs were operated to re-rank all the attributes according to the attribute importance evaluation functions, i.e. ‘CorrelationAttributeEval’, ‘GainRatioAttributeEval’, ‘InfoGainAttributeEval’, ‘OneRAttributeEval’, ‘ReliefFAttributeEval’, ‘SymmetricalUncertAttributeEval’, and ‘SVMAttributeEval’ in WEKA, combined with ‘Ranker’ search method. The latter attribute selection method is named ‘CfsSubsetEval’, running with ‘BestFirst’ searching method to pick out the best first attributes for classification (Figure 2).

#### Classifiers

Twenty-five classifiers were tested using WEKA, aiming to find the most suitable classifier in discriminating LGGs from HGGs as well as classifying WHO grade II, III and IV gliomas. Since the number of grade I glioma samples was too small (i.e. only three patients), they were not included in the following investigation. The details of each WEKA classifier applied in this study were given in Table 2. The classification accuracy and the area under the curve (AUC) were focused to compare the classification performance of different classification methods.

#### Cross-validation

The leave-one-out cross validation (LOOCV) strategy, which is widely used in machine learning studies and allows the use of most training data, was applied to assess the performance of each classifier in our study [18, 33]. Assuming the sample number is *N*, *N*-1 samples were selected as training data to construct the classifying model while the remained one sample was used as the testing data to testify the predicting accuracy. This operation would run *N* times and the summarized performance indicators of the classifiers were estimated after the whole validation procedure.

#### Model parameter

Parameter selection can have a significant influence on the performance of classifiers to some extent. Selecting appropriate model parameters can optimize the discriminative ability of the grading model. In WEKA, default parameter values or options were given, while the classifiers may reach their optimal performance by adjusting some critical parameters. Taking support vector machine (SVM) for example, four kernel types can be adopted, i.e. linear kernel, RBF kernel, polynomial kernel and sigmoid kernel, with additional predominant parameters for different SVM models such as *c* (penalty coefficient) for all models, *gamma* (radius of the kernel function) for RBF and sigmoid kernel SVM, *degree* for polynomial kernel SVM, etc. The general idea of parameter selection is to determine the optimal combination from a group of parameter combinations.

## SUPPLEMENTARY MATERIALS FIGURES AND TABLES









## Abbreviations

AIF: arterial input function
ASL: arterial spin labeling
AUC: area under the curve
BAT: bolus arrived time
BP: blood plasma
CBF: cerebral blood flow
DCE: dynamic contrast enhanced
DWI: diffusion weighted imaging
EES: extracellular extravascular space
FLAIR: fluid attenuated inversion recovery
FOV: field of view
GLCM: gray-level co-occurrence matrix
GLRLM: gray-level run-length matrix
GLSZM: gray-level size zone matrix
HGG: high-grade glioma
IQR: inter-quartile range
IVIM: intra-voxel incoherent motion
LGG: low-grade glioma
LOOCV: leave-one-out cross validation
MRI: magnetic resonance imaging
NEX: number of excitation
RFE: recursive feature elimination
ROC: receiver operating characteristics
SE-mean: standard error of mean
SMOTE: synthetic minority over-sampling technique
SVM: support vector machine
T1ce: contrast enhanced T1WI
T1WI: T1-weighted spin-echo imaging
TTP: time to peak
VOI: volume of interest
WEKA: Waikato environment for knowledge analysis
WHO: World Health Organization.

## References

[1] Wu CC, Guo WY, Chen MH, Ho DM, Hung AS, Chung HW (2012). Direct measurement of the signal intensity of diffusion-weighted magnetic resonance imaging for preoperative grading and treatment guidance for brain gliomas. J Chin Med Assoc.

[2] Arevalo-Perez J, Peck KK, Young RJ, Holodny AI, Karimi S, Lyo JK (2015). Dynamic contrast-enhanced perfusion MRI and diffusion-weighted imaging in grading of gliomas. J Neuroimaging.

[3] Li X, Zhu Y, Kang H, Zhang Y, Liang H, Wang S, Zhang W (2015). Glioma grading by microvascular permeability parameters derived from dynamic contrast-enhanced MRI and intratumoral susceptibility signal on susceptibility weighted imaging. Cancer Imaging.

[4] Zacharaki EI, Kanas VG, Davatzikos C (2011). Investigating machine learning techniques for MRI-based classification of brain neoplasms. Int J Comput Assist Radiol Surg.

[5] Law M, Yang S, Wang H, Babb JS, Johnson G, Cha S, Knopp EA, Zagzag D (2003). Glioma grading: sensitivity, specificity, and predictive values of perfusion MR imaging and proton MR spectroscopic imaging compared with conventional MR imaging. AJNR Am J Neuroradiol.

[6] Macyszyn L, Akbari H, Pisapia JM, Da X, Attiah M, Pigrish V, Bi Y, Pal S, Davuluri RV, Roccograndi L, Dahmane N, Martinez-Lage M, Biros G (2016). Imaging patterns predict patient survival and molecular subtype in glioblastoma via machine learning techniques. Neuro Oncol.

[7] Gao F, Guo R, Hu XJ, Li CJ, Li M (2015). Noninvasive tumor grading of glioblastomas before surgery using arterial spin labeling. A cohort study. Anal Quant Cytopathol Histpathol.

[8] Noguchi T, Yoshiura T, Hiwatashi A, Togao O, Yamashita K, Nagao E, Shono T, Mizoguchi M, Nagata S, Sasaki T, Suzuki SO, Iwaki T, Kobayashi K (2008). Perfusion imaging of brain tumors using arterial spin-labeling: correlation with histopathologic vascular density. AJNR Am J Neuroradiol.

[9] Togao O, Hiwatashi A, Yamashita K, Kikuchi K, Mizoguchi M, Yoshimoto K, Suzuki SO, Iwaki T, Obara M, Van Cauteren M, Honda H (2016). Differentiation of high-grade and low-grade diffuse gliomas by intravoxel incoherent motion MR imaging. Neuro Oncol.

[10] Hu YC, Yan LF, Wu L, Du P, Chen BY, Wang L, Wang SM, Han Y, Tian Q, Yu Y, Xu TY, Wang W, Cui GB (2014). Intravoxel incoherent motion diffusion-weighted MR imaging of gliomas: efficacy in preoperative grading. Sci Rep.

[11] Hu YC, Yan LF, Sun Q, Liu ZC, Wang SM, Han Y, Tian Q, Sun YZ, Zheng DD, Wang W, Cui GB (2017). Comparison between ultra-high and conventional mono b-value DWI for preoperative glioma grading. Oncotarget.

[12] Brynolfsson P, Nilsson D, Henriksson R, Hauksson J, Karlsson M, Garpebring A, Birgander R, Trygg J, Nyholm T, Asklund T (2014). ADC texture--an imaging biomarker for high-grade glioma?. Med Phys.

[13] Ryu YJ, Choi SH, Park SJ, Yun TJ, Kim JH, Sohn CH (2014). Glioma: application of whole-tumor texture analysis of diffusion-weighted imaging for the evaluation of tumor heterogeneity. PLoS One.

[14] Zhang N, Zhang L, Qiu B, Meng L, Wang X, Hou BL (2012). Correlation of volume transfer coefficient Ktrans with histopathologic grades of gliomas. J Magn Reson Imaging.

[15] Zollner FG, Emblem KE, Schad LR (2012). SVM-based glioma grading: optimization by feature reduction analysis. Z Med Phys.

[16] Emblem KE, Pinho MC, Zollner FG, Due-Tonnessen P, Hald JK, Schad LR, Meling TR, Rapalino O, Bjornerud A (2015). A generic support vector machine model for preoperative glioma survival associations. Radiology.

[17] Fehr D, Veeraraghavan H, Wibmer A, Gondo T, Matsumoto K, Vargas HA, Sala E, Hricak H, Deasy JO (2015). Automatic classification of prostate cancer Gleason scores from multiparametric magnetic resonance images. Proc Natl Acad Sci U S A.

[18] Inano R, Oishi N, Kunieda T, Arakawa Y, Yamao Y, Shibata S, Kikuchi T, Fukuyama H, Miyamoto S (2014). Voxel-based clustered imaging by multiparameter diffusion tensor images for glioma grading. Neuroimage Clin.

[19] Svolos P, Tsolaki E, Kapsalaki E, Theodorou K, Fountas K, Fezoulidis I, Tsougos I (2013). Investigating brain tumor differentiation with diffusion and perfusion metrics at 3T MRI using pattern recognition techniques. Magn Reson Imaging.

[20] Emblem KE, Due-Tonnessen P, Hald JK, Bjornerud A, Pinho MC, Scheie D, Schad LR, Meling TR, Zoellner FG (2014). Machine learning in preoperative glioma MRI: survival associations by perfusion-based support vector machine outperforms traditional MRI. J Magn Reson Imaging.

[21] LeCun Y, Bengio Y, Hinton G (2015). Deep learning. Nature.

[22] Roy B, Awasthi R, Bindal A, Sahoo P, Kumar R, Behari S, Ojha BK, Husain N, Pandey CM, Rathore RK, Gupta RK (2013). Comparative evaluation of 3-dimensional pseudocontinuous arterial spin labeling with dynamic contrast-enhanced perfusion magnetic resonance imaging in grading of human glioma. J Comput Assist Tomogr.

[23] Choi HS, Kim AH, Ahn SS, Shin NY, Kim J, Lee SK (2013). Glioma grading capability: comparisons among parameters from dynamic contrast-enhanced MRI and ADC value on DWI. Korean J Radiol.

[24] Chen HL, Yang B, Wang G, Wang SJ, Liu J, Liu DY (2012). Support vector machine based diagnostic system for breast cancer using swarm intelligence. J Med Syst.

[25] Parmar C, Grossmann P, Bussink J, Lambin P, Aerts HJ (2015). Machine learning methods for quantitative radiomic biomarkers. Sci Rep.

[26] Huang YQ, Liang CH, He L, Tian J, Liang CS, Chen X, Ma ZL, Liu ZY (2016). Development and validation of a radiomics nomogram for preoperative prediction of lymph node metastasis in colorectal cancer. J Clin Oncol.

[27] Liu ZC, Yan LF, Hu YC, Sun YZ, Tian Q, Nan HY, Yu Y, Sun Q, Wang W, Cui GB (2017). Combination of IVIM-DWI and 3D-ASL for differentiating true progression from pseudoprogression of Glioblastoma multiforme after concurrent chemoradiotherapy: study protocol of a prospective diagnostic trial. BMC Med Imaging.

[28] Sahoo P, Gupta PK, Awasthi A, Pandey CM, Patir R, Vaishya S, Saha I, Gupta RK (2016). Comparison of actual with default hematocrit value in dynamic contrast enhanced MR perfusion quantification in grading of human glioma. Magn Reson Imaging.

[29] Kang Y, Choi SH, Kim YJ, Kim KG, Sohn CH, Kim JH, Yun TJ, Chang KH (2011). Gliomas: histogram analysis of apparent diffusion coefficient maps with standard- or high-b-value diffusion-weighted MR imaging--correlation with tumor grade. Radiology.

[30] Mohanty AK, Senapati MR, Beberta S, Lenka SK (2013). Texture-based features for classification of mammograms using decision tree. Neural Comput Appl.

[31] Thibault G, Angulo J, Meyer F (2014). Advanced statistical matrices for texture characterization: application to cell classification. IEEE Trans Biomed Eng.

[32] Liu F, Wee CY, Chen H, Shen D (2014). Inter-modality relationship constrained multi-modality multi-task feature selection for Alzheimer's Disease and mild cognitive impairment identification. Neuroimage.

[33] Liu F, Guo W, Fouche JP, Wang Y, Wang W, Ding J, Zeng L, Qiu C, Gong Q, Zhang W, Chen H (2015). Multivariate classification of social anxiety disorder using whole brain functional connectivity. Brain Struct Funct.

