# Supplementary material for: Optimizing a machine learning based glioma grading system using multi-parametric MRI histogram and texture features
Source: Oncotarget. 2017 May 18;8(29):47816–30. doi: 10.18632/oncotarget.18001 (PMC5564607; doi:10.18632/oncotarget.18001)
Supplement: Supplementary file 1 [file oncotarget-08-47816-s001.pdf]

Optimizing a machine learning based glioma grading system using multi-parametric MRI histogram and texture features

SUPPLEMENTARY MATERIALS

(A)

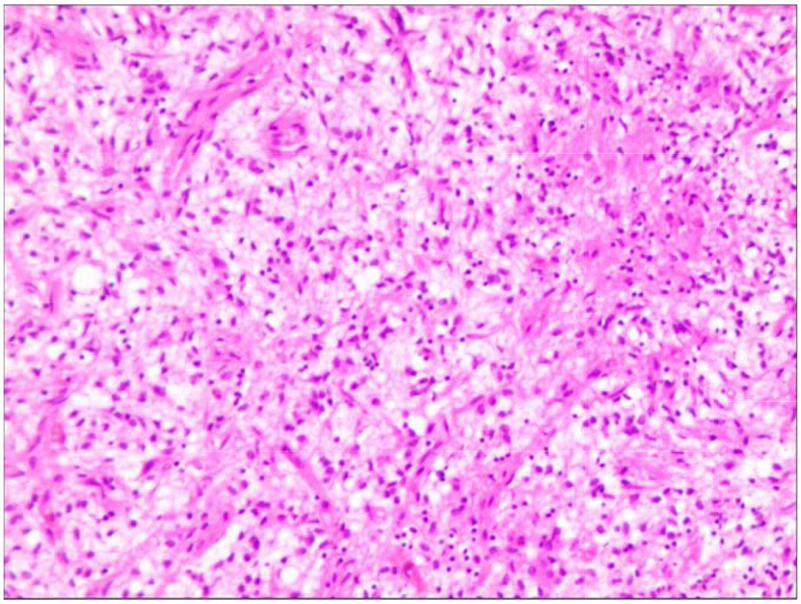

(B)

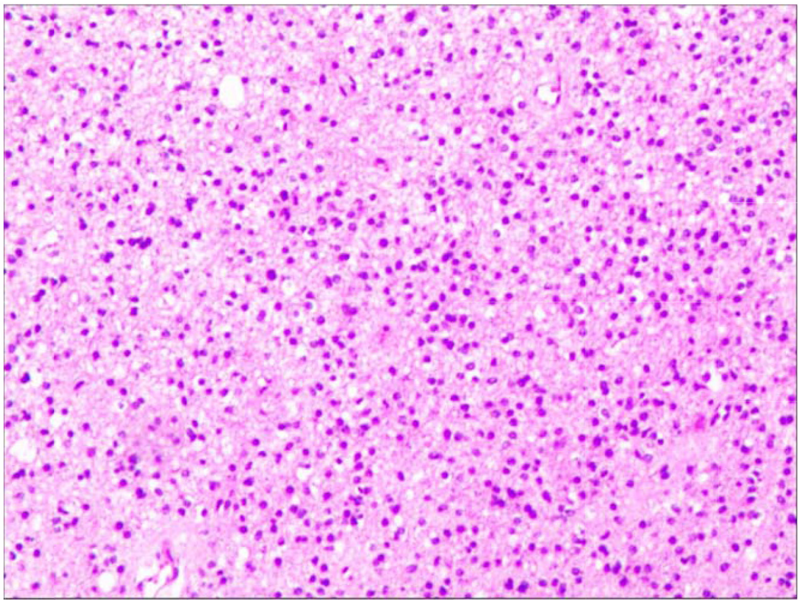

Supplementary Figure 1: The haematoxylin and eosin **(H&E)** results of individual grade I~IV glioma patients **(A~D)** in Figure 2. **(A)** A two-years old boy diagnosed of grade I glioma. **(B)** A 39-years old man diagnosed of grade II glioma. (*Contiuned*)

(C)

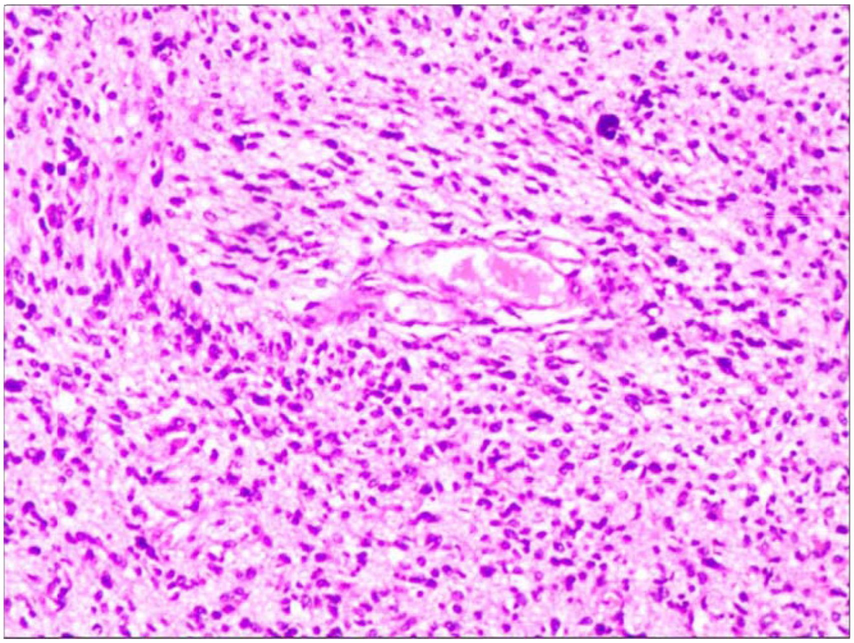

(D)

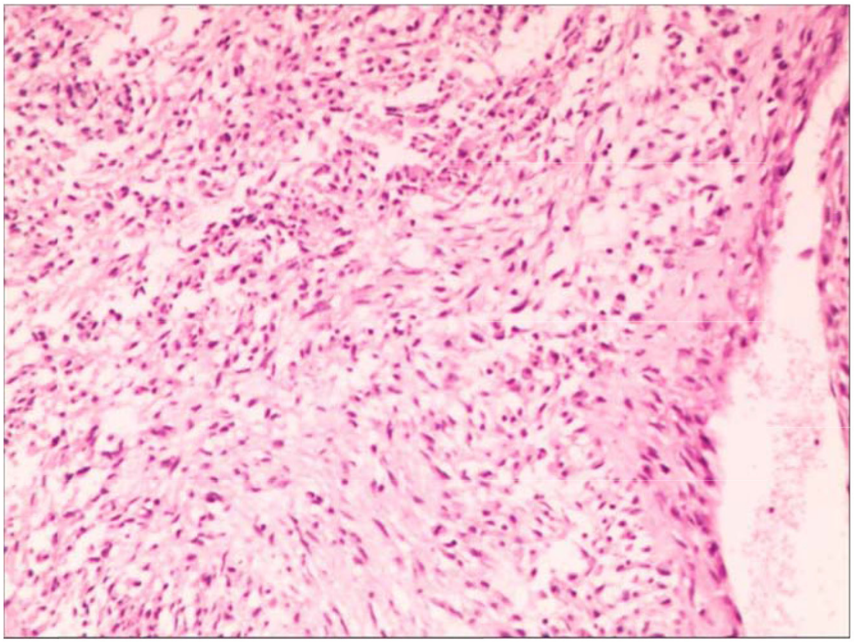

Supplementary Figure 1: (*Contiuned*) The haematoxylin and eosin (H&E) results of individual grade **I–IV** glioma patients **(A–D) in Figure 2. (C)** A 49-years old man diagnosed of grade III glioma. **(D)** A 61-years old women diagnosed of grade IV glioma.

Supplementary Table 1: The number of varied pathological types for gliomas with grade I–IV, respectively

Grade of glioma (number)	Pathological type (number)
Grade I (3)	Ganglioglioma (1)<br>Pilocytic astrocytoma (2)
Grade II (25)	Diffuse astrocytoma (11)<br>Oligodendroglioma (2)<br>Oligoastrocytoma (11)<br>Pleomorphic yellow astrocytoma (1)
Grade III (29)	Anaplastic astrocytoma (5)<br>Anaplastic oligodendroglioma (6)<br>Anaplastic oligoastrocytoma (18)
Grade IV (63)	Glioblastoma (62)<br>Gliosarcoma (1)

Supplementary Table 2: The first top 50 attributes ranked by SVM-RFE method in LGG and HGG as well as grade II, III and IV gliomas classification, respectively.

See Supplementary File 1

Supplementary Table 3: Multi-parametric MRI histogram and texture attribute names.

See Supplementary File 2

Supplementary Table 4: Definition of texture attributes.

See Supplementary File 3
