# Supplementary material for: Optimizing a machine learning based glioma grading system using multi-parametric MRI histogram and texture features
Source: Oncotarget. 2017 May 18;8(29):47816–30. doi: 10.18632/oncotarget.18001 (PMC5564607; doi:10.18632/oncotarget.18001)
Supplement: Supplementary file 2 [file oncotarget-08-47816-s002.docx]

**Supplementary Table 2: The first top 50 attributes ranked by SVM-RFE method in LGG and HGG as well as grade II, III and IV gliomas classification, respectively**.

| Rank no. | **Parametric attribute name** | |
| --- | --- | --- |
|  | **LGG vs. HGG** | **Grade II, III, and IV** |
| 1 | Perfusion_Peak (Kurtosis) | AUC_FP_ (90^th^ percentile) |
| 2 | Fast_ADC (Skewness) | Perfusion_PATLAK_Peak (GLCM_Correlation^*^) |
| 3 | Incremental_K^ep^ (GLSZM_SZHGE^*^) | AUC_FP_ (Mean) |
| 4 | Extended_TOFT_K^ep^ (PVAPH) | Extended_TOFT_V_e_ (Global_Variance^*^) |
| 5 | PATLAK_K^trans^ (Global_Variance^*^) | Extended_TOFT_V_e_ (GLRLM_GLN^*^) |
| 6 | Incremental_V_e_ (PVAPH) | AUC_FP_ (Q3) |
| 7 | Fast_ADC (GLSZM_HGZE^*^) | Extended_TOFT_K_ep_ (PVAPH) |
| 8 | TOFT_K^trans^ (Global_Skewness^*^) | AUC_FP_ (95^th^ percentile) |
| 9 | Extended_TOFT_K^ep^ (IQR) | Perfusion_Peak (Kurtosis) |
| 10 | Extended_TOFT_V_e_ (GLCM_Entrophy^*^) | Fast_ADC (Skewness) |
| 11 | Slow_fractional_ADC (Mode) | Extended_TOFT_K_ep_ (IQR) |
| 12 | AUC_FP_ (GLCM_Correlation^*^) | Extended_TOFT_V_e_ (GLCM_Variance^*^) |
| 13 | Perfusion_PATLAK_Peak (Maximum) | Extended_TOFT_V_e_ (GLSZM_SZHGE^*^) |
| 14 | Perfusion_ModelMap (PVAPH) | AUC_FP_ (MTFP) |
| 15 | AUC_FP_ (Q3) | Perfusion_WashIn (GLSZM_SZHGE^*^) |
| 16 | CBF(Maximum) | AUC_FP_ (IQR) |
| 17 | Slow_ADC (GLRLM_LRE^*^) | Perfusion_BF (Maximum) |
| 18 | AUC_FP_ (90^th^ percentile) | PATLAK_V_p_ (GLSZM_SZLGE^*^) |
| 19 | PATLAK_V_p_ (GLSZM_LZE^*^) | Extended_TOFT_V_e_ (GLSZM_HGZE^*^) |
| 20 | AUC_FP_ (Std) | Fast_ADC (GLRLM_SRHGE^*^) |
| 21 | Perfusion_Washout (GLCM_Energy^*^) | Incremental_K_ep_ (GLSZM_HGZE^*^) |
| 22 | Incremental_K_ep_ (GLSZM_HGZE^*^) | Extended_TOFT_V_p_ (MTFP) |
| 23 | AUC_FP_ (95^th^ percentile) | AUC_FP_ (GLSZM_LGZE^*^) |
| 24 | Perfusion_ModelMap (Mode) | Extended_TOFT_V_p_ (Variance) |
| 25 | AUC_FP_ (IQR) | Extended_TOFT_K_ep_ (GLCM_Correlation^*^) |
| 26 | Chi-square (Global_Variance^*^) | Incremental_K_ep_ (GLSZM_SZHGE^*^) |
| 27 | Extended_TOFT_V_p_ (HPH) | Fast_ADC (Kurtosis) |
| 28 | Perfusion_ BAT (90^th^ percentile) | AUC_FP_ (GLRLM_LGRE^*^) |
| 29 | Fast_ADC (Maximum) | Perfusion_PATLAK_BAT(10^th^ percentile) |
| 30 | Perfusion_ModelMap (GLRLM_LRHGE^*^) | Extended_TOFT_V_e_ (GLRLM_SRHGE^*^) |
| 31 | Perfusion_PATLAK_BAT (90^th^ percentile) | AUC_FP_ (GLRLM_SRLGE^*^) |
| 32 | PATLAK_K^trans^ (GLSZM_GLN^*^) | AUC_FP_ (GLCM_Correlation^*^) |
| 33 | Perfusion_Washout (GLSZM_SZE^*^) | TOFT_K^trans^ (Global_Skewness^*^) |
| 34 | Extended_TOFT_K_ep_ (Variance) | Chi-square (Global_Kurtosis^*^) |
| 35 | Perfusion_WashIn (GLSZM_HGZE^*^) | Perfusion_BAT (90^th^ percentile) |
| 36 | Perfusion_WashOut (MLFP) | Chi-square (GLSZM_LZHGE^*^) |
| 37 | Perfusion_TTP (PVAPH) | Perfusion_PATLAK_Peak (Variance) |
| 38 | Perfusion_PATLAK_Peak (GLSZM_SZLGE^*^) | Slow_ADC (GLRLM_LRLGE^*^) |
| 39 | AUC_FP_ (MTFP) | Fast_fractional_ADC (Global_Variance^*^) |
| 40 | CBF (GLRLM_SRLGE^*^) | AUC_FP_ (Q1) |
| 41 | Fast_ADC (Kurtosis) | Perfusion_PATLAK_Peak (GLCM_Contrast^*^) |
| 42 | Fast_fractional_ADC (Global_Variance^*^) | TOFT_V_e_ (GLSZM_SZHGE^*^) |
| 43 | Slow_fractional_ADC (5^th^ percentile) | AUC_FP_ (Std) |
| 44 | Extended_TOFT_V_e_ (GLRLM_GLN^*^) | Perfusion_ModelMap (Mode) |
| 45 | CBF (GLSZM_GLN^*^) | Extended_TOFT_V_p_ (Std) |
| 46 | Perfusion_Peak (GLSZM_SZLGE^*^) | Slow_fractional_ADC (5^th^ percentile) |
| 47 | Slow_ADC (Minimum) | Fast_fractional_ADC (95^th^ percentile) |
| 48 | Extended_TOFT_Ktrans (Global_Skewness^*^) | Fast_ADC (GLRLM_HGRE^*^) |
| 49 | Fast_ADC (Minimum) | Perfusion_WashOut (Maximum) |
| 50 | Slow_ADC (GLRLM_LRLGE^*^) | AUC_FP_ (Maximum) |

Note: * represents texture attributes.
