# Supplementary material for: Optimizing a machine learning based glioma grading system using multi-parametric MRI histogram and texture features
Source: Oncotarget. 2017 May 18;8(29):47816–30. doi: 10.18632/oncotarget.18001 (PMC5564607; doi:10.18632/oncotarget.18001)
Supplement: Supplementary file 3 [file oncotarget-08-47816-s003.docx]

**Supplementary Table 3: Multi-parametric MRI histogram and texture attribute names**

| **MRI modality** | **Parameter name** | **Attribute name** | **Attribute**  **number** |
| --- | --- | --- | --- |
| **DCE-**  **MRI**  **(k=24)** | **TOFT_K^trans^** | **Histogram (23):**  **Mean**  **Median**  **Mode**  **Standard Deviation (Std)**  **Variance**  **Standard error of mean**  **Skewness**  **Kurtosis**  **Minimum**  **Maximum**  **IQR/Q1/Q3**  **The 10^th^/90^th^ percentile**  **The 5^th^/95^th^ percentile**  **The mean of top/low five percent data (MTFP/MLFP)**  **Energy**  **Entrophy**  **Histogram peak height (HPH)**  **Parameter value at peak height (PVAPH)**  **Texture (37):**  **Global:**  **Variance, Skewness, Kurtosis**  **GLCM:**  **Energy, Contrast, Entrophy, Homogeneity, Correlation, Sumaverage, Variance, Dissimilarity**  **GLRLM:**  **SRE, LRE, GLN, RLN, RP, LGRE, HGRE, SRLGE, SRHGE, LRLGE, LRHGE, GLV, RLV**  **GLSZM:**  **SZE, LZE, GLN, ZSN, ZP, LGZE, HGZE, SZLGE, SZHGE, LZLGE, LZHGE, GLV, ZSV** | **（24+5+1）×（23+37）=1800** |
|  | **TOFT_K^ep^** |  |  |
|  | **TOFT_V_e_** |  |  |
|  | **Extended_TOFT_K^trans^** |  |  |
|  | **Extended_TOFT_K^ep^** |  |  |
|  | **Extended_TOFT_V_e_** |  |  |
|  | **Extended_TOFT_V_p_** |  |  |
|  | **PATLAK_K^trans^** |  |  |
|  | **PATLAK_V_p_** |  |  |
|  | **Incremental_K^trans^** |  |  |
|  | **Incremental_K^ep^** |  |  |
|  | **Incremental_V_e_** |  |  |
|  | **AUC_AIF_** |  |  |
|  | **AUC_FP_** |  |  |
|  | **Perfusion_TTP (TOFT/Extended TOFT/Incremental/PATLAK)** |  |  |
|  | **Perfusion_BAT (TOFT/Extended TOFT/Incremental)** |  |  |
|  | **Perfusion_BF (TOFT/Extended TOFT/Incremental)** |  |  |
|  | **Perfusion_Peak (TOFT/Extended TOFT/Incremental)** |  |  |
|  | **Perfusion_PATLAK_BAT** |  |  |
|  | **Perfusion_PATLAK_BF** |  |  |
|  | **Perfusion_PATLAK_Peak** |  |  |
|  | **Perfusion_ModelMap (Incremental)** |  |  |
|  | **Perfusion_WashIn (Incremental)** |  |  |
|  | **Perfusion_WashOut (Incremental)** |  |  |
| **Multi-b values DWI**  **(k=5)** | **Slow_ADC (D)** |  |  |
|  | **Fast_ADC (D^*^)** |  |  |
|  | **Slow_fractional_ADC (fast f)** |  |  |
|  | **Fast_fractional_ADC (slow f)** |  |  |
|  | **Chi-square** |  |  |
| **3D-ASL**  **(k=1)** | **CBF** |  |  |
