# Supplementary material for: Optimizing a machine learning based glioma grading system using multi-parametric MRI histogram and texture features
Source: Oncotarget. 2017 May 18;8(29):47816–30. doi: 10.18632/oncotarget.18001 (PMC5564607; doi:10.18632/oncotarget.18001)
Supplement: Supplementary file 4 [file oncotarget-08-47816-s004.docx]

**Supplementary Table 4: Definition of texture attributes**

| **Input**: a volume of *V*(*x,y,z*) | | |
| --- | --- | --- |
| ***Texture model*** | ***Attribute name*** | ***Equation*** |
| **Global**  ***P***: the first-order histogram of *V*(*x,y,z*)  ***P*(*i*)** : the number of voxels with gray-level *i*.  ***N_g_***: the number of gray-level bins set for *P*. $p(i)\text{=}\frac{P(i)}{\sum_{i=1}^{N_{g}} P(i)}$  | Variance | $\sum_{i=1}^{N_{g}} (i-\mu)^{2}p(i)$ |
|  | Skewness | $\sigma^{-3}\sum_{i=1}^{N_{g}} (i-\mu)^{3}p(i)$ |
|  | Kurtosis | $\sigma^{-4}\sum_{i=1}^{N_{g}} \left[(i-\mu)^{4}p(i)\right]-3$ |
| **Gray-Level**  **Co-occurence Matrix (GLCM)**  ***P***: the GLCM of *V*(*x,y,z*)  ***P*(*i,j*)** : the number of times voxels with gray-level *i* were neighbors with voxels of gray-level *j* in *V*.  ***N_g_***: the pre-defined number of quantized gray-levels set in *V*. $p(i,j)\text{=}\frac{P(i,j)}{\sum_{i=1}^{N_{g}} \sum_{j=1}^{N_{g}} P(i,j)}$ $\mu_{i}=\sum_{i=1}^{N_{g}} i\sum_{j=1}^{N_{g}} p(i,j)$ $\mu_{j}=\sum_{j=1}^{N_{g}} j\sum_{i=1}^{N_{g}} p(i,j)$ $\sigma_{i}=\sum_{i=1}^{N_{g}} \text{（}i\text{-}\mu_{i}\text{）}^{2}\sum_{j=1}^{N_{g}} p(i,j)$ $\sigma_{j}=\sum_{j=1}^{N_{g}} \text{（}j\text{-}\mu_{j}\text{）}^{2}\sum_{i=1}^{N_{g}} p(i,j)$        | Energy | $\sum_{i=1}^{N_{g}} \sum_{j=1}^{N_{g}} \left[p(i,j)\right]^{2}$ |
|  | Contrast | $\sum_{i=1}^{N_{g}} \sum_{j=1}^{N_{g}} (i-j)^{2}p(i,j)$ |
|  | Correlation | $\sum_{i=1}^{N_{g}} \sum_{j=1}^{N_{g}} \frac{(i-\mu_{i})(j-\mu_{j})p(i,j)}{\sigma_{i}\sigma_{j}}$ |
|  | Homogeneity | $\sum_{i=1}^{N_{g}} \sum_{j=1}^{N_{g}} \frac{p(i,j)}{1+\left\vert i-j\right\vert}$ |
|  | Variance | $\frac{1}{N_{g}\times N_{g}}\sum_{i=1}^{N_{g}} \sum_{j=1}^{N_{g}} \left[(i-\mu_{i})^{2}p(i,j)+(j-\mu_{j})^{2}p(i,j)\right]$ |
|  | Sum Average | $\frac{1}{N_{g}\times N_{g}}\sum_{i=1}^{N_{g}} \sum_{j=1}^{N_{g}} \left[ip(i,j)+jp(i,j)\right]$ |
|  | Entrophy | $\sum_{i=1}^{N_{g}} \sum_{j=1}^{N_{g}} p(i,j)\log_{2}(p(i,j))$ |
|  | Dissimilarity | $\sum_{i=1}^{N_{g}} \sum_{j=1}^{N_{g}} \left\vert i-j\right\vert p(i,j)$ |
| **Gray-Level Run-Length Matrix (GLRLM)**  ***P***: the GLRLM of *V*(*x,y,z*)  ***P*(*i,j*)**: the number of runs of gray-level *i* and of length *j* in *V*.  ***N_g_***: the pre-defined number of quantized gray-levels set in *V*.  ***L_r_***: the length of the longest run (of any gray-level) in *V*. $p(i,j)\text{=}\frac{P(i,j)}{\sum_{i=1}^{N_{g}} \sum_{j=1}^{L_{r}} P(i,j)}$ $\mu_{i}=\sum_{i=1}^{N_{g}} i\sum_{j=1}^{L_{r}} p(i,j)$ $\mu_{j}=\sum_{j=1}^{L_{r}} j\sum_{i=1}^{N_{g}} p(i,j)$    | Short Run Emphasis (SRE) | $\sum_{i=1}^{N_{g}} \sum_{j=1}^{L_{r}} \frac{p(i,j)}{j^{2}}$ |
|  | Long Run Emphasis (LRE) | $\sum_{i=1}^{N_{g}} \sum_{j=1}^{L_{r}} j^{2}p(i,j)$ |
|  | Gray-Level Nonuniformity (GLN) | $\sum_{i=1}^{N_{g}} \left(\sum_{j=1}^{L_{r}} p(i,j)\right)^{2}$ |
|  | Run-Length Nonuniformity (RLN) | $\sum_{j=1}^{L_{r}} \left(\sum_{i=1}^{N_{g}} p(i,j)\right)^{2}$ |
|  | Run Percentage (RP) | $\frac{\sum_{i=1}^{N_{g}} \sum_{j=1}^{L_{r}} p(i,j)}{\sum_{j=1}^{L_{r}} j\sum_{i=1}^{N_{g}} p(i,j)}$ |
|  | Low Gray-Level Run Emphasis (LGRE) | $\sum_{i=1}^{N_{g}} \sum_{j=1}^{L_{r}} \frac{p(i,j)}{i^{2}}$ |
|  | High Gray-Level Run Emphasis (HGRE) | $\sum_{i=1}^{N_{g}} \sum_{j=1}^{L_{r}} i^{2}p(i,j)$ |
|  | Short Run Low Gray-Level Emphasis (SRLGE) | $\sum_{i=1}^{N_{g}} \sum_{j=1}^{L_{r}} \frac{p(i,j)}{i^{2}j^{2}}$ |
|  | Short Run High Gray-Level Emphasis (SRHGE) | $\sum_{i=1}^{N_{g}} \sum_{j=1}^{L_{r}} \frac{i^{2}p(i,j)}{j^{2}}$ |
|  | Long Run Low Gray-Level Emphasis (LRLGE) | $\sum_{i=1}^{N_{g}} \sum_{j=1}^{L_{r}} \frac{j^{2}p(i,j)}{i^{2}}$ |
|  | Long Run High Gray-Level Emphasis (LRHGE) | $\sum_{i=1}^{N_{g}} \sum_{j=1}^{L_{r}} i^{2}j^{2}p(i,j)$ |
|  | Gray-Level Variance (GLV) | $\frac{1}{N_{g}\times L_{r}}\sum_{i=1}^{N_{g}} \sum_{j=1}^{L_{r}} (ip(i,j)-\mu_{i})^{2}$ |
|  | Run-Length Variance (RLV) | $\frac{1}{N_{g}\times L_{r}}\sum_{i=1}^{N_{g}} \sum_{j=1}^{L_{r}} (jp(i,j)-\mu_{j})^{2}$ |
| **Gray-Level Size Zone Matrix (GLSZM)**  ***P***: the GLSZM of *V*(*x,y,z*)  ***P*(*i,j*)**: the number of 3D zones of gray-level *i* and size *j* in *V*.  ***N_g_***: the pre-defined number of quantized gray-levels set in *V*.  ***L_z_***: the size of the largest zone (of any gray-level) in *V*. $p(i,j)\text{=}\frac{P(i,j)}{\sum_{i=1}^{N_{g}} \sum_{j=1}^{L_{\text{z}}} P(i,j)}$ $\mu_{i}=\sum_{i=1}^{N_{g}} i\sum_{j=1}^{L_{z}} p(i,j)$ $\mu_{j}=\sum_{j=1}^{L_{z}} j\sum_{i=1}^{N_{g}} p(i,j)$    | Small Zone Emphasis (SZE) | $\sum_{i=1}^{N_{g}} \sum_{j=1}^{L_{z}} \frac{p(i,j)}{j^{2}}$ |
|  | Large Zone Emphasis (LZE) | $\sum_{i=1}^{N_{g}} \sum_{j=1}^{L_{z}} j^{2}p(i,j)$ |
|  | Gray-Level Nonuniformity (GLN) | $\sum_{i=1}^{N_{g}} \left(\sum_{j=1}^{L_{z}} p(i,j)\right)^{2}$ |
|  | Zone-Size Nonuniformity (ZSN) | $\sum_{j=1}^{L_{z}} \left(\sum_{i=1}^{N_{g}} p(i,j)\right)^{2}$ |
|  | Zone Percentage (ZP) | $\frac{\sum_{i=1}^{N_{g}} \sum_{j=1}^{L_{z}} p(i,j)}{\sum_{j=1}^{L_{z}} j\sum_{i=1}^{N_{g}} p(i,j)}$ |
|  | Low Gray-Level Zone Emphasis (LGZE) | $\sum_{i=1}^{N_{g}} \sum_{j=1}^{L_{z}} \frac{p(i,j)}{i^{2}}$ |
|  | High Gray-Level Zone Emphasis (HGZE) | $\sum_{i=1}^{N_{g}} \sum_{j=1}^{L_{z}} i^{2}p(i,j)$ |
|  | Small Zone Low Gray-Level Emphasis (SZLGE) | $\sum_{i=1}^{N_{g}} \sum_{j=1}^{L_{z}} \frac{p(i,j)}{i^{2}j^{2}}$ |
|  | Small Zone High Gray-Level Emphasis (SZHGE) | $\sum_{i=1}^{N_{g}} \sum_{j=1}^{L_{z}} \frac{i^{2}p(i,j)}{j^{2}}$ |
|  | Large Zone Low Gray-Level Emphasis (LZLGE) | $\sum_{i=1}^{N_{g}} \sum_{j=1}^{L_{z}} \frac{j^{2}p(i,j)}{i^{2}}$ |
|  | Large Zone High Gray-Level Emphasis (LZHGE) | $\sum_{i=1}^{N_{g}} \sum_{j=1}^{L_{z}} i^{2}j^{2}p(i,j)$ |
|  | Gray-Level Variance (GLV) | $\frac{1}{N_{g}\times L_{z}}\sum_{i=1}^{N_{g}} \sum_{j=1}^{L_{z}} (ip(i,j)-\mu_{i})^{2}$ |
|  | Zone-Size Variance (ZSV) | $\frac{1}{N_{g}\times L_{z}}\sum_{i=1}^{N_{g}} \sum_{j=1}^{L_{z}} (jp(i,j)-\mu_{j})^{2}$ |
